# Combined surgery and sclerotherapy for 13 years: a case report of a patient with CLOVES

**DOI:** 10.3389/fped.2024.1336358

**Published:** 2024-03-04

**Authors:** Shiqi Wang, Siming Yuan

**Affiliations:** Department of Plastic Surgery, Jinling Hospital, Affiliated Hospital of Medical School, Nanjing University, Nanjing, China

**Keywords:** CLOVES syndrome, vascular malformation, truncal mass, overgrowth syndrome, sclerotherapy

## Abstract

Congenital lipomatous overgrowth, vascular malformations, epidermal nevi, and skeletal anomalies (CLOVES) constitute a rare overgrowth disorder resulting from a mosaic function-acquiring mutation in the *PIK3CA* gene. Targeted drugs for the PI3K-AKT signaling pathway remain under clinical trial and surgery is commonly used to meet both aesthetic and functional requirements for CLOVES patients. We report here the course and experience of a male patient treated at our institution for up to 13 years. The course of treatment consisted of nine anhydrous ethanol sclerotherapy procedures and two segmental trunk mass resections. After undergoing sequential treatment, the patient experienced improved thoracic deformity and scoliosis, enabling him to grow and develop normally.

## Introduction

CLOVES syndrome was first identified in 2009 (1), derived from the acronym for four distinguishing conditions: congenital lipomatous overgrowth, vascular malformations, epidermal nevi, and scoliosis/skeletal/spinal anomalies. Its pathogenesis is attributed to a post-zygotic, somatic mutation in *PIK3CA* gene, involved in the receptor tyrosine kinase phosphatidylinositol 3-kinase (PI3)-AKT growth signaling pathway (2). In 2015, it was classified as a more severe form of *PIK3CA*-related Overgrowth syndrome (PROS) (3). To aid in the diagnosis, it is crucial to consider the patient's medical history alongside the presence of somatic *PIK3CA* gene mutations (e.g., hotspot mutations PIK3CA codons 542, 545, and 1,047) (4). The treatment guidelines for CLOVES syndrome are currently still being developed and refined. Clinical trials are currently being conducted on inhibitors that target individual molecules of the PI3K-AKT pathway, such as sirolimus (rapamycin, mTOR inhibitor) (5), Alpesilib (BYL719) (6) that targets p110α, and miransertib (MK-7075, previously ARQ092, an AKT inhibitor) (7). However, these inhibitors are still in their trial stages and have been used only sympathetically in critically ill patients. Currently, surgeons in plastic surgery often resort to surgical excision when faced with patients with CLOVES syndrome. Due to the large size, surgical excision of overgrowth masses often poses a significant challenge to medical staff (8). Minimally invasive sclerotherapy is a common treatment for low-flow vascular malformations (9).

We present the case of a 17-year-old male CLOVES syndrome patient who initially consulted our department at the age of 4 and received a formal diagnosis in 2015. Exome sequencing revealed a missense mutation at the *PIK3CA* mutation locus c.1258T>C in affected somatic cells, with a mutation frequency of 18.6%.

## Case description

**Figure 1:**
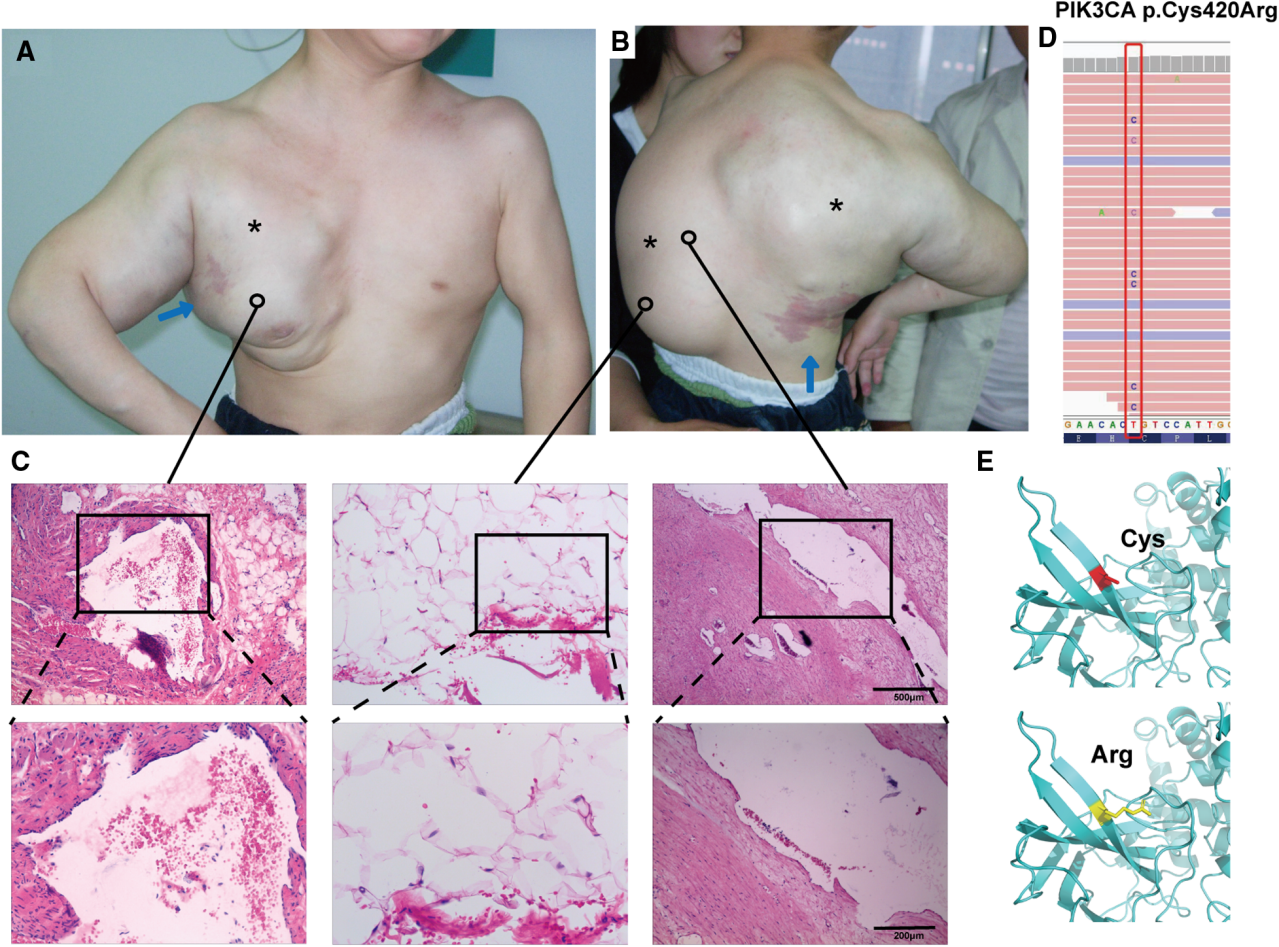
Clinical features and mutation analysis of our patient. The patient has a large mass on the thoracic back, right axilla, and upper extremities (*; **A,B**), and the child has an irregular port-wine stain on the left chest near the axilla, and another on the right lower part of the back (blue arrows; **A,B**). Histopathology showed variable-sized vascular tissue with fibrous tissue hyperplasia and degeneration of the walls, with erythrocytes and lymphoid fluid-like material visible in the lumen (**C**, left and right panels), and pathology showed subcutaneous adipose tissue neoplastic hyperplasia (**C**, middle panel) (hematoxylin-eosin stain; magnification ×40, local image magnification ×100). Mutational analysis of affected skin revealed the presence of the heterozygous missense mutation Cys420Arg in *PIK3CA*, which is absent in blood (**D**). Three-dimensional prediction shows changes in protein structure before and after mutation (**E**).

A 4-year-old male child presented to the Department of Plastic Surgery of the Jinling Hospital with a congenital large swelling on the thoracic back, right axilla and upper limb. The child was born at term without any special circumstances and was the first child of a non-consanguineous relative in China. The swelling has been present since birth and has gradually increased in size over time. It tends to rapidly worsen in size and cause discomfort following a cold. The swelling elicits tenderness and pain when palpated, but no pulse can be detected, and the skin temperature is normal. Moreover, there is an irregular port-wine stain on the left chest near the axilla, and another on the right lower part of the back. A smooth fatty-overgrowth mass is visible on the left side of the lower back (Figures 1A,B).

On the CT scan, it was observed that there was a mass present in the right upper extremity of the child. The borders of the mass were poorly defined and there were uneven internal densities with mostly fluid densities. There were also fat-density shadows present on the left side of the child's back. The right side of the thorax appeared to be extruded and deformed. The enhanced CT showed significant enhancement of the solid portion in the venous and delayed phases.

**Figure 2:**
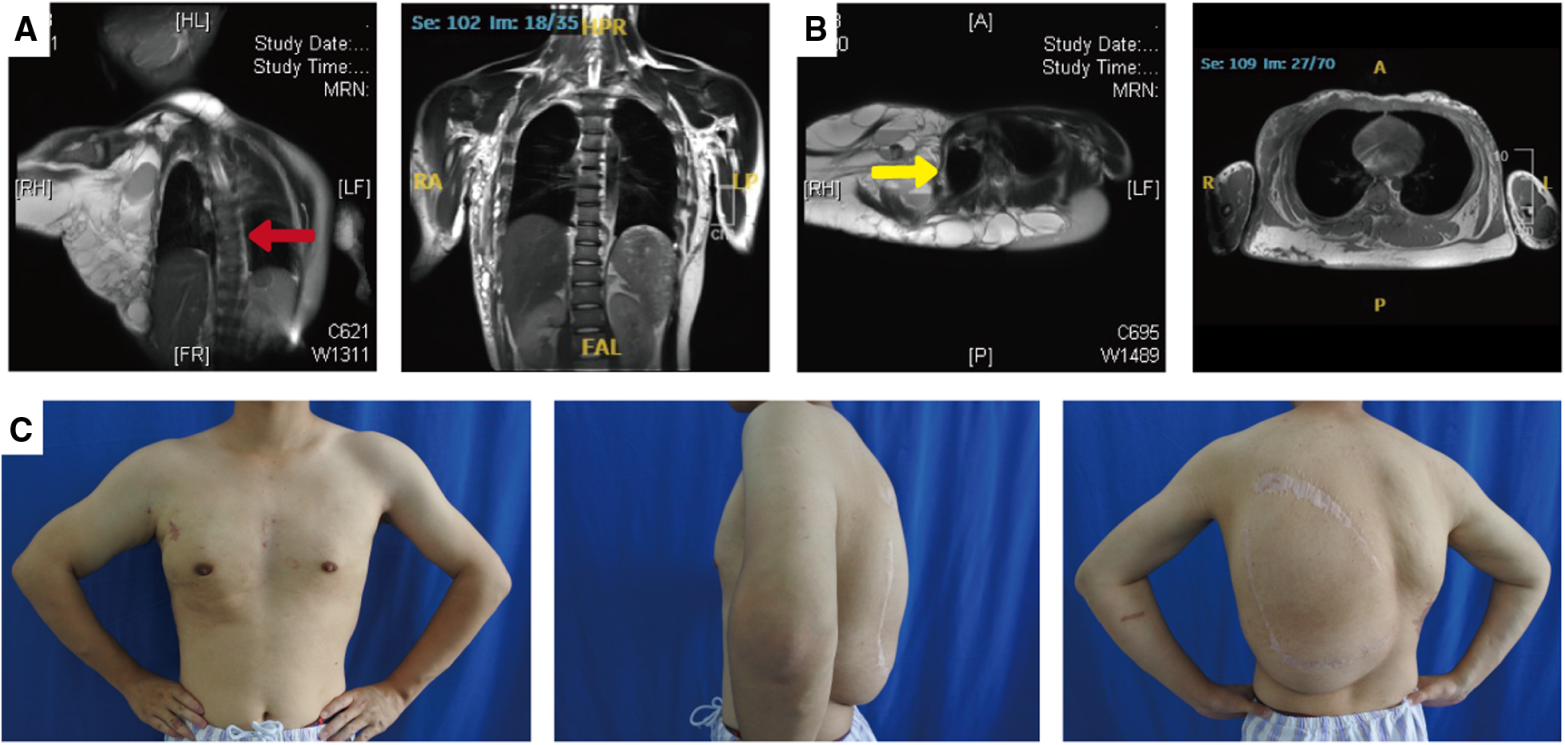
Photographs and MRI images of the patient's spine and thorax before and after treatment. Magnetic resonance imaging showed scoliosis (**A**; red arrow) and thoracic compression deformity (**B**; yellow arrow). After treatment, the patient has a normal spine (**A**, right panel) and a symmetrical thorax (**B**, right panel). The patient's arms and chest have a nearly normal appearance, and the back mass has subsided significantly (**C**).

Magnetic resonance imaging showed clear results: the left coronal and transverse views revealed multiple, variable-sized, thin-walled cystic long T1 and long T2 signals in the right shoulder, dorsum, and left axilla. The capsule was rich in plasma. Short T1 isotropic T2 signals were visible in the right axilla. Both T1WI and T2WI showed high signals, which were associated with hemorrhage. The enhancement scan reveals a marked improvement in the cyst wall. The child has scoliosis (red arrow; Figure 2A) and thoracic compression deformity (yellow arrow; Figure 2B).

During the examination, a color Doppler test was performed, revealing a cystic mass encompassing both arterial and venous blood flow. Additionally, an abdominal ultrasound detected another cystic mass in the right adrenal area that subsequently disappeared during follow-up without special treatment.

The hematology report upon admission showed that the child had mild hypochromic anemia, likely due to iron deficiency, and mild hypoproteinemia. It is worth noting that the child exhibited an elevated D-dimer, potentially indicating a coagulopathy. The child received a clinical diagnosis of CLOVES syndrome in 2015. The adipose tissue from a mass on the child's back underwent whole exome sequencing, revealing a missense mutation c.1258T>C in the *PIK3CA* gene (Figure 1D), which is absent in blood, resulting in the substitution of cysteine with arginine in position 420 of the protein crystal structure (Figure 1E). Other pathogenic mutations screened by exome sequencing are listed in Supplementary Table S1.

## Surgical management and sclerotherapy

**Figure 3:**
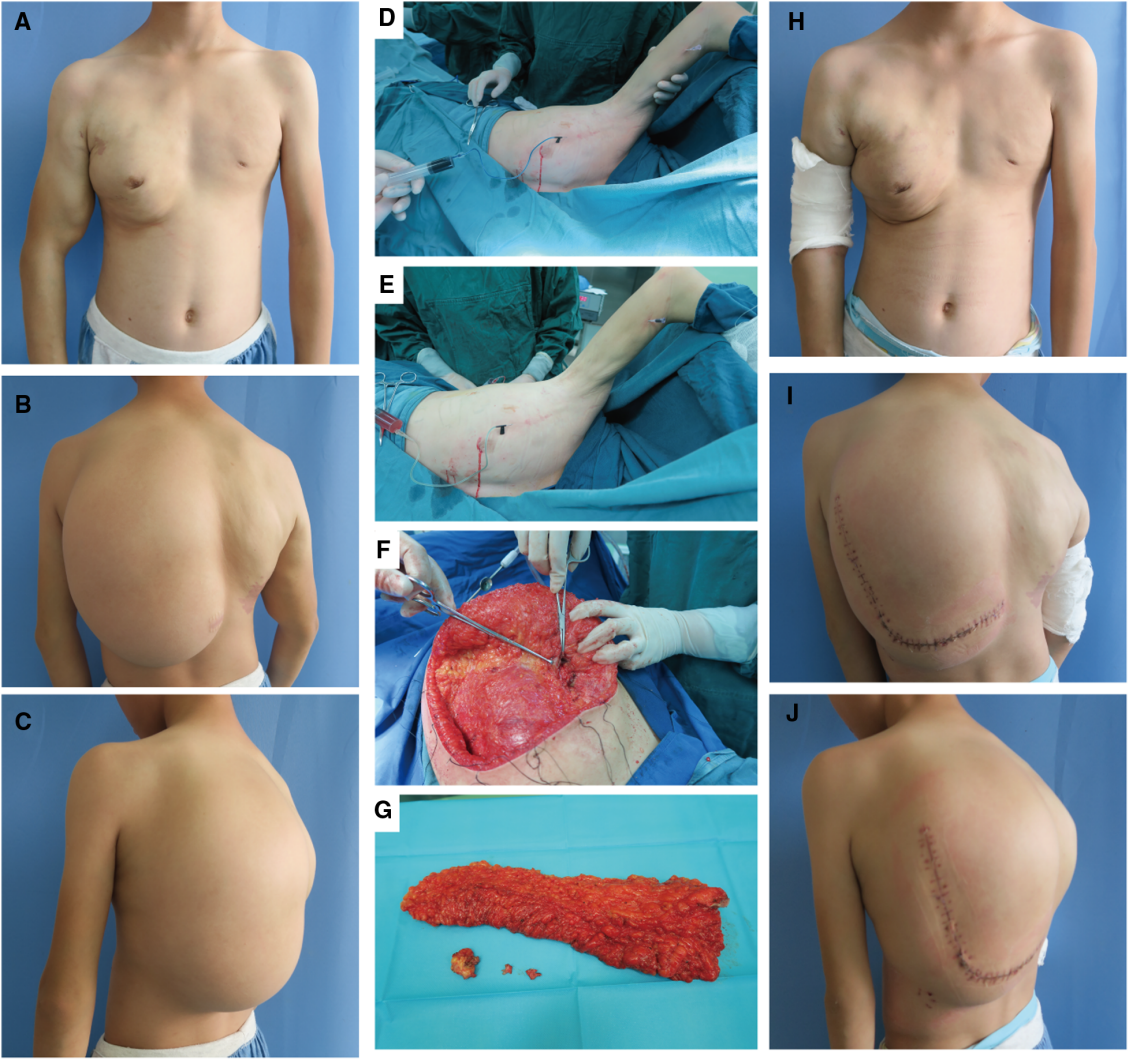
Surgery-related photographs of our patient. Preoperative frontal, lateral, and dorsal photographs of the patient (**A–C**). Intraoperative demonstration of the trunk mass as an adipose tissue-encapsulated giant lymphatic malformation (**D**) Excised trunk mass (**E**) Sclerotherapy with methylene blue tracing and lymphatic fluid extraction (**F,G**). Frontal, lateral and dorsal photographs of the patient after resection of the trunk mass combined with sclerotherapy (**H–J**).

The patient underwent ten admissions between 2010 and 2023, the timeline of which is shown in Table 1. Except for the first admission, where only anti-inflammatory treatment was administered, sclerotherapy for giant lymphatic malformation in the thoracic dorsum and the right upper limb was conducted on the remaining nine occasions. Additionally, two left dorsal lipoma resections were carried out in 2016 and 2017, respectively. Figure 3 presents the pre and post-operative photographs of the patient's initial trunk mass resection coupled with sclerotherapy intervention, along with intraoperative images.

**Table 1:** Analysis of surgical treatment timeline and perioperative details in the CLOVES patient.

Age (years old)	Surgery	Sclerosing agent	Pathology specimen weight (g)
4	–		–
8	Sclerotherapy	Absolute ethane (124 ml)	–
9	Sclerotherapy	Absolute ethane (244 ml)	–
10	Sclerotherapy/surgical excision	Absolute ethane (154 ml)	825
11	Sclerotherapy/surgical excision	Absolute ethane (144 ml)	563
12	Sclerotherapy	Absolute ethane (174 ml)	341
12	Sclerotherapy	Absolute ethane (124 ml)	–
13	Sclerotherapy	Absolute ethane (104 ml)	–
14	Sclerotherapy	Absolute ethane (124 ml)	–
17	Sclerotherapy	Absolute ethane (124 ml)	–

The basic procedure of sclerotherapy is as follows (Figures 3D–E): puncture with a syringe attached to an injection needle in the lesion area, retraction of lymphatic fluid in the lesion and injection of anhydrous alcohol solution at multiple sites. The amount of anhydrous ethanol sclerosing agent used ranged between 10 and 24 ml per session. Subsequent to two treatments, a considerable reduction in the vascular deformity of the child's right upper limb was observed (Figures 3A vs. H).

The left dorsal hyperplastic lipectomy was bifurcated into two resections, the upper one-third and the lower two-thirds, with consideration to the patient's tolerance. Magnetic resonance indicated the tumor-encapsulated vascular system in the left dorsal mass prior to the initial resection. During the operation, after removing up to the deep fascial layer, large malformed lymphatic vessels could be visualized (Figure 3F). The postoperative pathology examination of the excised tissue affirmed the presence of subcutaneous adipose tissue tumor-like hyperplasia and vascular malformations (Figure 1C). The excised specimens weighed 341 and 825 grams, respectively. Analysis of the surgical treatment timeline and perioperative details can be found in Table 1.

## Follow-up

The child was followed up every 3 months. After treatment, the child's thorax changed from a right-sided giant complex vascular malformation with compression and collapse to a left-right symmetrical and basically normal one (Figures 2A,C); the scoliosis deformity improved significantly (Figures 2B,C). Upon undergoing mass excision and sclerotherapy, the volume of lipomatous hyperplasia was reduced (Figure 2C). Although the lipomatous hyperplasia still grew slowly, it did not have a considerable impact on the person's quality of life.

## Discussion

In this case report, we describe a case of CLOVES with up to 13 years of follow-up. Lipectomy and sclerotherapy were employed with positive outcomes, enabling normal growth and development of the thorax and spine during childhood.

In 2007, Sapp et al. (10) reported on patients with congenital lipomatous overgrowth and vascular problems. Malformations and epidermal nevi presented unique symptoms and were named CLOVE syndrome. Later, Alomari et al. (1) suggested expanding the name to CLOVES to emphasize the association with scoliosis, bone, and spine abnormalities. Through sequencing, the study confirmed that CLOVES syndrome arises due to a somatic *PIK3CA* mutation occurring during early embryonic development. The detection of the same mutant allele at all sites indicates early-onset development and subsequent effect on multiple cell lineages (2). The existence of somatic gene mutations is deemed essential evidence for the definitive diagnosis of CLOVES syndrome (3).

Targeted agents directed against various targets of the PI3K-AKT pathway are also considered to have therapeutic potential. Sirolimus (an mTOR inhibitor of rapamycin), a targeted agent for breast cancer, was first shown to be effective and relatively safe in inhibiting disease progression in a 61-case phase II clinical trial (5). However, further investigations revealed that Sirolimus only partially inhibits mTORC1 and has no impact on mTORC2, resulting in weak mTORC1 inhibition triggering negative feedback, amplifying upstream signaling, and ultimately facilitating AKT signaling (7). In 2018, for palliative use in patients, Alpesilib (BYL719) proved to be more effective than rapamycin at slowing down the progression of the disease (6). PROS patients benefit from the inhibition of AKT rather than mTOR. Therefore, clinical development of the pan-AKT inhibitor miransertib (MK-7075, formerly ARQ092) is underway in PROS patients (7). However, all current drugs are in clinical trials, and in most hospitals, plastic surgeons still employ surgery to achieve aesthetic and functional recovery by removing the mass (8).

In this case, the patient initially received a diagnosis of giant mixed vascular malformation, as CLOVES syndrome had not yet been defined when he began treatment with us at age 4. It was not until 2015 that the diagnosis was officially revised to CLOVES syndrome. We observed an intriguing phenomenon where the mass in our patient underwent rapid growth during periods of cold and fever, which is a typical response of the lymphatic system in the case of an infection. The child had undergone multiple sclerotherapy and trunk mass resections since childhood, which significantly alleviated their thorax and spine.

Anhydrous alcohol was used as the sclerosing agent during the sclerotherapy. Anhydrous ethanol has a long-standing and prevalent use in the sclerotherapy of lymphangiovenous malformations and hemangiomas. Although studies indicate that sclerosing agents, like polydocanol and bleomycin, have a less severe effect, anhydrous ethanol is deemed to be the most efficient, with the lowest recurrence rate (9, 11). The patient, in this case, responded positively to the sclerotherapy with anhydrous ethanol and did not experience any adverse effects (12).

The patient's coagulation suggests mild hypercoagulation, which is commonly observed in CLOVES syndrome (13, 14). The prethrombotic state is linked with the depletion of coagulation factors caused by abnormal venous stasis. This depletion is also due to thrombosis, which is supported by raised D-dimer levels, reduced fibrinogen levels, extended prothrombinase time, and mild thrombocytopenia (15). Furthermore, CLOVES syndrome has an increased risk of developing pulmonary thromboembolism (14). The dilated veins are connected to the deep venous system, serving as reservoirs of coagulation factor depletion and foci of thrombosis, as well as direct sources of pulmonary thromboemboli (13). These findings suggest prompt anticoagulation, particularly before surgery.

At 4 years old, the patient underwent an ultrasound which indicated a cystic mass in the right adrenal region. However, during follow-up, the cystic mass disappeared without further treatment. Although it has been ruled out that the patient has a urological malignancy, it is worth noting that patients with CLOVES have a higher likelihood of developing Wilms tumor (2, 16–18), a malignant embryonal renal tumor associated with hemihypertrophy and certain overgrowth disorders. Wilms tumor may result from the presence of *PIK3CA* mutant cells within somatic cells at the kidney site of individuals with CLOVES (16). We concur with the guidelines advocating the consideration of quarterly abdominal ultrasound screenings until 7 years of age for children with CLOVES syndrome (3).

In conclusion, surgical reduction and minimally invasive therapies can be utilized to help CLOVES children return to normal growth, and it is essential to minimize disease progression and functional disability to avoid devastating complications such as spinal cord invasion and consequent spinal cord compression and paralysis.

## Data Availability

The original contributions presented in the study are included in the article/Supplementary Material, further inquiries can be directed to the corresponding author.

## References

[1] Alomari AI. Characterization of a distinct syndrome that associates complex truncal overgrowth, vascular, and acral anomalies: a descriptive study of 18 cases of CLOVES syndrome. Clin Dysmorphol. (2009) 18(1):1–7. 10.1097/MCD.0b013e328317a716 19011570

[2] Kurek KC Luks VL Ayturk UM Alomari AI Fishman SJ Spencer SA Somatic mosaic activating mutations in PIK3CA cause CLOVES syndrome. Am J Hum Genet. (2012) 90(6):1108–15. 10.1016/j.ajhg.2012.05.006 22658544 PMC3370283

[3] Keppler-Noreuil KM Rios JJ Parker VE Semple RK Lindhurst MJ Sapp JC PIK3CA-related overgrowth spectrum (PROS): diagnostic and testing eligibility criteria, differential diagnosis, and evaluation. Am J Med Genet A. (2015) 167a(2):287–95. 10.1002/ajmg.a.36836 25557259 PMC4480633

[4] Youssefian L Vahidnezhad H Baghdadi T Ghaznavi A Li Q Tabrizi M Fibroadipose hyperplasia versus Proteus syndrome: segmental overgrowth with a mosaic mutation in the PIK3CA gene. J Invest Dermatol. (2015) 135(5):1450–3. 10.1038/jid.2015.15 25602158

[5] Adams DM Trenor CC 3rd Hammill AM Vinks AA Patel MN Chaudry G Efficacy and safety of sirolimus in the treatment of complicated vascular anomalies. Pediatrics. (2016) 137(2):e20153257. 10.1542/peds.2015-3257 26783326 PMC4732362

[6] Venot Q Blanc T Rabia SH Berteloot L Ladraa S Duong JP Targeted therapy in patients with PIK3CA-related overgrowth syndrome. Nature. (2018) 558(7711):540–6. 10.1038/s41586-018-0217-9 29899452 PMC7610773

[7] Ranieri C Di Tommaso S Loconte DC Grossi V Sanese P Bagnulo R In vitro efficacy of ARQ 092, an allosteric AKT inhibitor, on primary fibroblast cells derived from patients with PIK3CA-related overgrowth spectrum (PROS). Neurogenetics. (2018) 19(2):77–91. 10.1007/s10048-018-0540-1 29549527 PMC5956072

[8] Weissler JM Shubinets V Carney MJ Low DW. Complex truncal masses in the setting of CLOVES syndrome: aesthetic and functional implications. Aesthetic Plast Surg. (2017) 41(3):591–9. 10.1007/s00266-016-0771-1 28032156

[9] Yang C Li M Li X Zhu J Shu C. Foam sclerotherapy in the treatment of hemangiomas and venous malformations. Dermatol Surg. (2023) 49(9):855–61. 10.1097/DSS.0000000000003857 37432998

[10] Sapp JC Turner JT van de Kamp JM van Dijk FS Lowry RB Biesecker LG. Newly delineated syndrome of congenital lipomatous overgrowth, vascular malformations, and epidermal nevi (CLOVE syndrome) in seven patients. Am J Med Genet A. (2007) 143a(24):2944–58. 10.1002/ajmg.a.32023 17963221

[11] Lee BB Kim DI Huh S Kim HH Choo IW Byun HS New experiences with absolute ethanol sclerotherapy in the management of a complex form of congenital venous malformation. J Vasc Surg. (2001) 33(4):764–72. 10.1067/mva.2001.112209 11296330

[12] Do YS Yakes WF Shin SW Lee B-B Kim D-I Liu WC Ethanol embolization of arteriovenous malformations: interim results. Radiology. (2005) 235(2):674–82. 10.1148/radiol.2352040449 15858106

[13] Reis J 3rd Alomari AI Trenor CC 3rd Adams DM Fishman SJ Spencer SA Pulmonary thromboembolic events in patients with congenital lipomatous overgrowth, vascular malformations, epidermal nevi, and spinal/skeletal abnormalities and Klippel-Trénaunay syndrome. J Vasc Surg Venous Lymphat Disord. (2018) 6(4):511–6. 10.1016/j.jvsv.2018.01.015 29909856

[14] Alomari AI Burrows PE Lee EY Hedequist DJ Mulliken JB Fishman SJ. CLOVES syndrome with thoracic and central phlebectasia: increased risk of pulmonary embolism. J Thorac Cardiovasc Surg. (2010) 140(2):459–63. 10.1016/j.jtcvs.2010.04.023 20537357

[15] Oduber CEU Gerdes VEA van der Horst CMAM Bresser P. Vascular malformations as underlying cause of chronic thromboembolism and pulmonary hypertension. J Plast Reconstr Aesthet Surg. (2009) 62(5):684–9. 10.1016/j.bjps.2007.12.062 18571489

[16] Michel ME Konczyk DJ Yeung KS Murillo R Vivero MP Hall AM Causal somatic mutations in urine DNA from persons with the CLOVES subgroup of the PIK3CA-related overgrowth spectrum. Clin Genet. (2018) 93(5):1075–80. 10.1111/cge.13195 29231959 PMC5899663

[17] Peterman CM Fevurly RD Alomari AI Trenor CC 3rd Adams DM Vadeboncoeur S Sonographic screening for wilms tumor in children with CLOVES syndrome. Pediatr Blood Cancer. (2017) 64(12):e26684. 10.1002/pbc.26684 28627003

[18] Peterman CM Vadeboncoeur S Mulliken JB Fishman SJ Liang MG. Wilms tumor screening in diffuse capillary malformation with overgrowth and macrocephaly-capillary malformation: a retrospective study. J Am Acad Dermatol. (2017) 77(5):874–8. 10.1016/j.jaad.2017.06.014 28822558

