# Supplementary material for: Combined surgery and sclerotherapy for 13 years: a case report of a patient with CLOVES
Source: Front Pediatr. 2024 Mar 4;12:1336358. doi: 10.3389/fped.2024.1336358 (PMC10944971; doi:10.3389/fped.2024.1336358)
Supplement: Supplementary file 1 [file Table1.docx]

Table S1. Other potential pathogenic mutations in patients screened by whole exome sequencing.

| **Hugo Symbol** | **Allele Frequency** | **cDNA Change** | **Protein Change** | **COSMIC ID** |
| --- | --- | --- | --- | --- |
| *CFAP47* | 0.12 | c.8386G>A | p.D2796N | COSV66215765 |
| *SMPDL3B* | 0.024 | c.886G>A | p.A296T | COSV65855392 |
| *IL1F10* | 0.036 | c.428G>A | p.R143H | COSV61750504 |
| *MMP1* | 0.047 | c.13C>T | p.P5S | COSV59511868 |
| *LENG8* | 0.033 | c.1821G>A | p.R607R | COSV58730791 |
| *OGFOD3* | 0.032 | c.169G>A | p.A57T | COSV57342189 |
| *PCDHGA2* | 0.027 | c.e1+35015C>T |  | COSV54036058 |
| *NCKAP1L* | 0.067 | c.e21-11A>T |  | COSV53207245 |
| *SLC27A1* | 0.024 | c.587C>T | p.T196M | COSV53101714 |
| *EPB41L5* | 0.091 | c.e5-9A>T |  | COSV105846103 |
| *NFXL1* | 0.107 | c.1294C>T | p.R432C | COSV105198887 |
| *ENDOG* | 0.055 | c.882G>A | p.A294A | COSV104669399 |
| *AVL9* | 0.129 | c.e6+67G>A |  | COSV100594852 |

COSMIC: Catalogue of Somatic Mutations in Cancer databases.
